# Poorer Well-Being in Children With Misophonia: Evidence From the *Sussex Misophonia Scale for Adolescents*

**DOI:** 10.3389/fpsyg.2022.808379

**Published:** 2022-04-06

**Authors:** Louisa J. Rinaldi, Rebecca Smees, Jamie Ward, Julia Simner

**Affiliations:** School of Psychology, University of Sussex, Brighton, United Kingdom

**Keywords:** misophonia, sound-sensitivity, sensory sensitivity, aversion, wellbeing

## Abstract

**Objective:**

Misophonia is an unusually strong aversion to a specific class of sounds – most often human bodily sounds such as chewing, crunching, or breathing. A number of studies have emerged in the last 10 years examining misophonia in adults, but little is known about the impact of the condition in children. Here we set out to investigate the well-being profile of children with misophonia, while also presenting the first validated misophonia questionnaire for children.

**Materials and Methods:**

We screened 142 children (10–14 years; Mean 11.72 SD 1.12; 65 female, 77 male) using our novel diagnostic [the *Sussex Misophonia Scale for Adolescents* (*SMS-Adolescent*)]. This allowed us to identify a group of children already manifesting misophonia at that age – the first population-sampled cohort of child misophonics examined to date. Children and their parents also completed measures of well-being (for convergent validation of our SMS-Adolescent) and creative self-construct (for discriminant validation).

**Results:**

Data show that children with misophonia have significantly elevated levels of anxiety and obsessive compulsive traits. Additionally children with misophonia have significantly poorer life-satisfaction, and health-related quality of life. As predicted, they show no differences in creative self-construct.

**Conclusion:**

Together our data suggest the first evidence in population sampling of poorer life outcomes for children with misophonia, and provide preliminary convergent and discriminant validation for our novel misophonia instrument. Our data suggest a need for greater recognition and therapeutic outlets for adolescents with misophonia.

## Introduction

Misophonia is a disorder of decreased tolerance to certain classes of sounds, which trigger unusual negative emotions such as anger, disgust, or anxiety (Swedo et al., 2022). Typical triggers include everyday sounds such as chewing, crunching, clicking, or breathing. These sounds are not particularly loud, and easily ignored by most other people, but can be highly aversive to people with misophonia (for reviews see Potgieter et al., 2019; Williams et al., 2021). The condition may be associated with subtle organisational differences in the brain that likely arise during development, and lead to important variations in sound tolerance – which can impact profoundly on daily life. People with misophonia show increased functional and structural connectivity in regions related to threat, emotion, and salience (Kumar et al., 2017; Schröder et al., 2019), suggesting that sounds are more prominent and emotionally distressing than they might be for most other people. Here we consider the roots of this condition, by seeking to better understand misophonia in children. Our study aims to identify how the condition can be recognised in young cohorts (10–14 years) using a novel instrument, and whether misophonia is associated with demonstrably poorer well-being across multiple measures.

Several case-studies have described children and adolescents who have sought treatment for misophonia in clinical environments (Johnson et al., 2013; Webber et al., 2014; Kamody and del Conte, 2017; Dover and McGuire, 2021). However, the present study is the first to explore misophonia in young samples who have *not* self-referred for study/treatment, and this can provide vital information. Clearly, children who are studied at clinic have already shown sufficient difficulties for their parents or caregivers to seek clinical support. Examining well-being in such environments is therefore circular (i.e., since children with poorer well-being are precisely those who seek treatment). Here we take a different approach by asking questions about well-being in a sample of children with misophonia who have not already sought treatment, and will almost certainly not even know that their misophonia is a recognised condition. Specifically, we screened a large sample of children to identify those with misophonia among them. As such, this is the first ever study of a population-sampled cohort of child misophonics, and we give details below of how our participants were identified.

Some studies suggest as many as 20% of the population may have some degree of misophonia (Wu et al., 2014; Zhou et al., 2017) with yet-higher rates in groups with elevated anxiety (Naylor et al., 2020) but potentially lower rates cross-culturally (Zhou et al., 2017; Kılıç et al., 2021). However, the exact prevalence may still be unknown since it is difficult to draw a line between everyday disliking, and the type of disliking linked to misophonia (e.g., most people dislike messy eating-sounds but only misophonics will feel the extreme emotions that make tolerating these sounds almost impossible). It is therefore important to use a robust methodology when identifying people with misophonia for research purposes. Although several statistically-tested misophonia questionnaires exist for adults (Wu et al., 2014; Rinaldi et al., 2021; Rosenthal et al., 2021; Vitoratou et al., 2021), there are no validated tests for children. Our review found that child-completed (or indeed parent-completed) assessments of any kind are rare in misophonia, and those that exist are typically “add-ons” to adult diagnostics (e.g., with instructions to substitute “my sound issues” for “my child’s sound issues”). This sometimes create ambiguous items [e.g., *My (→ my child’s) sound issues currently make me unhappy*; Who is unhappy: parent or child?] or require parents to comment on difficult-to-distinguish internal mental states in their children (e.g., *My child feels helpless? Or isolated? Or guilty?*). Therefore, a second aim of this study was to validate a novel diagnostic of misophonia in children: our newly devised *Sussex Misophonia Scale for Adolescents* (SMS-Adolescent). We describe this briefly below.

Our adolescent misophonia measure is based on an existing scale (Sussex Misophonia Scale; Rinaldi et al., 2021) we recently produced for adults. Importantly, we created this original adult questionnaire in such a way as to be ideally suited for adapting to adolescents, by using psycholinguistic norming data to ensure its language was appropriate not just for adults but also for children (see section “Materials and Methods”). Additionally, the original adult questionnaire was devised to be time-efficient (e.g., for when testing adults in large cohorts or within a battery of other tests) but this also makes it suitable for the shorter attention spans of younger participants. Finally, the adult questionnaire was specifically written in such a way that a parallel adolescent measure could be created in the future with only the most minimal adaptation; specifically, it would require only a single word change in just four items exchanging *work* for *school* (e.g., *I avoid work* → *I avoid school*; see Appendix for full adolescent questionnaire). Hence, our original adult questionnaire was ideally suited to be adapted into an adolescent version, which we have done in the current study. We then administered this questionnaire to a sample of children 10–14 years, to identify those with misophonia, whom we could simultaneously examine for well-being.

Any research study – and indeed any diagnostic – of misophonia in adolescents would be especially valuable for a number of reasons. Misophonia was named and recognized only recently (Jastreboff and Jastreboff, 2001) and has not yet entered formal diagnostic manuals such as the DSM-5 and ICD-11 (American Psychiatric Association, 2013; World Health Organization, 2020). This lack of widespread recognition has partly contributed to the relatively poorer life-outcomes reported by people with misophonia – especially those with more profound aversions. Here we look at similar outcomes in children, testing constructs that have been examined in the adult literature. In adults, misophonia has been linked with poorer well-being, where quality of life declines with increasing misophonia symptoms (Jager et al., 2020) while depressive symptoms increase (Eijsker et al., 2019), and where people with misophonia show higher rates of anxiety and obsessive compulsive disorder (OCD)/obsessive symptoms (Cusack et al., 2018). However, far less is known about misophonia in children, even though the condition appears to arise at some point during childhood or adolescence (Rouw and Erfanian, 2018; Lewin et al., 2021). Moreover, misophonia can potentially worsen with age if left unaddressed, and give rise to coping strategies (e.g., wearing headphones) that could theoretically worsen sensitivity over time (Palumbo et al., 2018). Importantly, young children often cannot advocate for themselves to seek treatment. And even if they do so, a lack of clinical and research understanding means that medical professionals are often unable to provide children with the support they need. Our aim therefore is to demonstrate how to recognise the presentation of misophonia in children, and to examine its impact on well-being.

To understand the focus of our research on well-being, we must understand that “well-being” is a broad construct (Pollard and Lee, 2003), incorporating different concepts such as life satisfaction (Diener, 2000), hedonic well-being (e.g., emotional stability, good mental health), eudaemonic well-being (e.g., positive mental attitude, fulfillment; Ryff et al., 1995), bodily/health-related well-being (e.g., Erhart et al., 2009), and the psychological/physical/social well-being that contributes to health-related quality-of-life (The Whoqol Group, 1998; Erhart et al., 2009). As we might therefore expect, the literature on childhood well-being is also extremely heterogeneous, focussing on both single well-being concepts, and multi-dimensional ones (Pollard and Lee, 2003; Ben-Arieh and Frønes, 2007; McLellan and Steward, 2015; Casas, 2019; Newland et al., 2019). Importantly however, differences in children’s well-being predict inequalities in a number of different ways. For example, lower levels of well-being have been linked to lower educational attainment (Sammons et al., 2008; Lindeboom et al., 2010; Morrison Gutman and Vorhaus, 2012), school exclusions (Parry-Langdon et al., 2008), poorer behaviour (Sylva et al., 2008), and lowered life chances (Cornaglia et al., 2015). And well-being is known to be particularly poor in children with sensory differences (e.g., higher rates of anxiety in children with multi-sensory sensitivities and synaesthesia; Simner et al., 2021). It is therefore important to understand the well-being profiles of children with misophonia, including areas of anxiety and OCD/obsessive symptoms.

In summary, our research aims to understand the well-being of adolescents with misophonia, with a primary focus on anxiety and OCD/obsessive symptoms, given that these have shown misophonia-linked associations in adults (Schröder et al., 2013; Wu et al., 2014; Cusack et al., 2018; Naylor et al., 2020). A secondary focus is on the well-being elements of health-related quality of life, and satisfaction with life, both predicted to decline in misophonia as they do in a range of other conditions (e.g., schizophrenia; Chang et al., 2011; Fervaha et al., 2016). A final aim of our manuscript is to validate a novel diagnostic measure for adolescent misophonia (our SMS-Adolescent). If our misophonia scale successfully identifies a group of children who go on to show significant differences from their peers *in other ways* (i.e., in well-being), we suggest this goes some way toward validating the measure itself. To be clear, an ideal approach to validation might include other procedures such as examining the Receiver Operator Characteristics (ROC) of our instrument (see Mehdi and Ahmadi, 2011); doing this in our adult questionnaire has allowed us to show that the adult measure is an “excellent” tool for separating a large group of pre-identified misophonics from a large group of pre-identified controls (Rinaldi et al., 2021). In children however, we do not have a “large group of pre-identified misophonics” – for precisely the reasons we are conducting this research. In other words, we have a problem of circularity: the lack of diagnostics and poor recognition for childhood misophonia means there are few or no large cohorts of child misophonics we could use to validate any diagnostic with ROC analyses. Therefore, we instead seek *convergent* validity, showing that children identified as having misophonia by the SMS-Adolescent are also those showing broader well-being deficits, compared to their peers. We will therefore screen a cohort of children for misophonia using our adolescent misophonia measure (SMS-Adolescent) and then explore the well-being of those identified as having misophonia (see section “Materials and Methods”).

Finally, we also aim to validate our questionnaire via *discriminant* validity, by demonstrating that our construct of interest (misophonia, as identified by our novel questionnaire) does not correlate with unrelated constructs where we would not expect it to. For this we selected a measure of creative self-concept, in which we asked our child-participants to evaluate how well they felt they performed in creative subjects at school. Creative self-concept is a robust construct of creativity that has been well studied (McKay et al., 2017; Snyder et al., 2020) and correlates with direct measurements of creative activities and achievements. We hypothesise that children with misophonia should score no differently to controls in creative self-concept. This would provide some evidence of discriminant validity for our misophonia questionnaire (the SMS-Adolescent), in addition to convergent validity from our well-being measures.

## Materials and Methods

### Participants

We tested 275 participants, comprising 142 children and adolescents aged 10–14 years (Mean 11.72 SD 1.12; 65 female, 77 male), along with 133 of their parents (113 female, 19 male, 1 prefer not to say) whose children had a mean age 11.73 (SD 1.14; 64 female, 69 male; There were nine more children than adults since nine families ended testing after the child-measures but before the adult-measures. We therefore included these families in our analyses of child-measure only).

Our participants were drawn from the MULTISENSE project, a large-scale random screening study focussing on multiple aspects of childhood development (e.g., multisensory processing, creativity, and attainment; e.g., Simner et al., 2021). The inclusion criteria for the MULTISENSE project was to be in Years 2–5 within 22 Infant and Primary schools across Sussex in the south of England in 2016, where uptake for the study was 99% and the sample comprised over 3,000 children in the initial recruitment wave. As an indicator of affluence/poverty (Taylor, 2018) the mean school-level *Free School Meal* percentage for this cohort was 13.44%, where the national average from the same year is 14.5%, and our schools ranged in FSM status from 0.7 to 38.1%. The 142 children in our current study were those whose parents had agreed to stay in touch for future screening,^1^ and they were tested for the current study 4 years after initial recruitment. (Parent) participants were entered into a £100 prize draw. This study was approved by the Sussex University Science and Technology Ethics Committee (reference number ER/LR290/3).

### Materials and Procedure

Testing took place between November 2020 and March 2021. Participants completed our study from home, using our in-house web application, which houses tests and advice on misophonia.^2^ Parent participants were sent a URL via email to take part, and this led them directly into our testing page without any access to the broader framework. The study began with a request for demographic information on age, gender etc. Participants then completed our six measures shown below; the first measure below was completed by parents and the subsequent five were completed by children.

#### The Screen for Childhood Anxiety Related Disorders

The Screen for Childhood Anxiety Related Disorders (SCARED; Birmaher et al., 1997, 1999) is a parent-completed 41-item questionnaire which screens for anxiety symptoms. Scores measure overall anxiety, with additional sub-scales of *Panic Disorder, General Anxiety, Separation Anxiety, Social anxiety, and School Avoidance*. Questions are presented as statements, which parents rate based on their child over the past 3 months. For example, Item 7 relates to generalised anxiety and states *My child is nervous*. Parents respond on a 3-point Likert scale: *Not true or hardly ever true*/*Somewhat true or sometimes true*/*Very true or often true*. This widely used measure is reliable in a number of ways, including in terms of internal consistency (α = 0.93), test–retest reliability, and parent–child agreement (Birmaher et al., 1997, 1999). In our own sample we found excellent internal consistency (α = 0.95). This questionnaire took approximately 5 min to complete.

#### Sussex Misophonia Scale for Adolescents

This novel self-report questionnaire presents 48 known misophonia triggers in Part 1 (see Table 1), and then 39 Likert-type statements in Part 2. In Part 1, participants were told that the questionnaire concerned things they hear and see, and they were asked: *Have you always hated these things? Or don’t you mind them?* Using check boxes, participants respond Yes/No to eight broad categories (e.g., *I hate*… *the sound of people eating*; see Table 1). If all eight responses were *No*, participants proceeded to Part 2. But for any *Yes* response, this revealed a full list of triggers within that category. For example, if participants responded *Yes* to *I hate the sound of people eating*, this revealed check boxes for eight types of eating-sound [*crunchy foods (e.g., apples); crispy snacks; chewing; lip smacking; swallowing; slurping (a drink); wet mouth sounds (e.g., yoghurt); and other eating sound*; see Table 1]. Across our eight categories, we presented a total of 48 trigger items, although our conditional logic allowed us to ask this in a time-efficient way. These 48 items were drawn from a detailed literature search, representing triggers identified for misophonia at the time of testing (see Rinaldi et al., 2021).

**TABLE 1:** Triggers for misophonia, and their superordinate category.

No.	We’re going to ask you about things you see and hear every day. Have you always hated these things? Or don’t you mind them? I hate…	Which do you hate hearing (or seeing, for category 7)? Tick all that apply.
1	The sound of people eating	Crunchy foods (e.g., Apples); crispy snacks; chewing; lip smacking; swallowing; slurping (a drink); wet mouth sounds (e.g., yoghurt); and other
2	The sound of repetitive tapping	Pen clicking; foot tapping/foot on floor; repetitive barking; tapping pen/pencil; tapping finger; typing on a computer; and other
3	The sound of rustling	Rustling paper; rustling plastic; and other
4	Throat sounds	Throat clearing; hiccups; humming; and other
5	Sounds people make through their mouth and nose	Breathing; snorting (e.g., when people laugh); nose sniffing; coughing; snoring; whistling; sneezing; burping; and other
6	Some voice sounds	Certain accents; some people’s voices; certain letter sounds; certain vowels; certain consonants; and other
7	Repetitive visual movements	Repetitive leg rocking; foot shuffling; people rocking back and forth on their chair; and other
8	Some background sounds (e.g., fridge humming)	Clock ticking; car engines; refrigerator humming; dishwasher; washing machine/dryer; fan; and other

*Categories are shown first; sub-set items are revealed in the event of a positive response. Note that seven out of eight trigger-categories are for sounds, while one category is non-auditory because people with misophonia can also be triggered by repetitive visual movements such as leg-swaying.*

In Part 2, participants were shown 39 statements, with the question: *How often do these things happen to you?* Responses were given on a 5-point scale (*Never*, *Rarely*, *Sometimes*, *Often*, and *Always*). Examples include: *Hatred of some sounds make me feel lonely* (Item 18); *I don’t do well at school because of distractions from sounds* (Item 12); *I want to get pay-back on people who make certain sounds* (Item 37); *I cover my ears to block out certain sounds* (Item 28); and *Sounds often cause me physical pain* (Item 9).^3^ We point out that questions related to pain might be suggestive of conditions such as hyperacusis (i.e., pain, discomfort, or a sense of “fullness” in the ears, especially from loud sounds). However, hyperacusis is co-morbid with misophonia (Jastreboff and Jastreboff, 2014), and these questions correlate highly with all others (Rinaldi et al., 2021). They are included here because they will alert clinicians to pain symptomology and the possible need for screening of other pain-related conditions.

This questionnaire was adapted from an almost identical version for adults (Rinaldi et al., 2021), with only a single-word difference, changing *work* to *school* in four items (Q12, Q14, Q22, and Q31; see Appendix). This was possible since the original adult version had been created in such a way as to be ideally suited to adapting for adolescents. Specifically, we had used psycholinguistic norming data to ensure its language was appropriate not just for adults but also for children. We conducted a linguistic analysis of its vocabulary using age-of-acquisition norms (Gilhooly and Logie, 1980; Bird et al., 2001) retrieved via the N-Watch psycholinguistics tool (Davis, 2005). This analysis showed that the vocabulary within this test makes it appropriate for adolescents in our study, having a mean age-of-acquisition of 3 years 9 months, with an upper age of 8 years 2 months (based on 122 of its 173 words, which were retrievable from N-Watch).

In total, Parts 1 and 2 contained 109 items, with 48 items revealed conditionally, meaning our questionnaire took only 5–10 min to complete. In part 2, our measure showed an excellent overall internal consistency of α = 0.97. Receiver Operator Characteristic additionally show this questionnaire to be an “excellent measure” for identifying misophonia in adults (see Rinaldi et al., 2021) and the current study will add validation for the adolescent version.

#### Very Short Wellbeing Questionnaire for Children

The Very Short Wellbeing Questionnaire for Children (VSWQ-C; Smees et al., 2020) questionnaire captures health-related quality-of-life in a measure for children aged 6+ years. Its four positively-worded questions are *Have you got on well in class*? *Have you got on well at home*? *Have you got on well with friends*?, and *Has your body felt well*? Children completed the questionnaire by rating statements on a 5-point Likert scale: *Never*, *Hardly ever*, *Sometimes*, *Mostly*, or *Always*. The VSWQ-C was developed from a consideration of the Health-Related Quality-of-life literature (e.g., Ravens-Sieberer and KIDSCREEN Group Europe, 2006; Solans et al., 2008) and designed for fast administration, while covering key levels of well-being (*home life*, *school life*, *friends*, and *health*). A recent validation on more than 1,500 children (Smees et al., 2020) shows the VSWQ-C to have excellent concurrent validity (*r* > 0.7) with longer measures such as the KIDSCREEN-10 (Ravens-Sieberer and KIDSCREEN Group Europe, 2006), suggesting it successfully taps into global well-being. The VSWQ-C was previously shown to have an internal consistency of α = 0.66 in children aged 9–10 years old, and in our sample had an internal consistency of α = 0.80.

#### Satisfaction With Life Scale-Child

The Satisfaction with Life Scale-Child (SWLS-C; Gadermann et al., 2010, 2011) is a 5-item measure for children and adolescents to self-report their life satisfaction. It is an adaptation of the adult *Satisfaction with Life Scale* (Diener et al., 1985), and children responded using a 5-point Likert scale (from *Disagree a lot* to *Agree a lot*). Its five items are: *In most ways my life is close to the way I would want it to be*; *The things in my life are excellent*; *I am happy with my life*; *So far I have gotten the important things I want in life*; and *If I could live my life over, I would have it the same way*. Gadermann et al. (2010, 2011) have successfully demonstrated the measure’s construct validity, and convergent and discriminant validity. They additionally reported an internal consistency of α = 0.86, and in our own sample we found an internal consistency of α = 0.90.

#### The Obsessive Compulsive Inventory – Child Version

The Obsessive Compulsive Inventory – Child Version (OCI-CV; Foa et al., 2010) is a 21-item child-report measure assessing obsessive compulsive symptoms in children and adolescents aged 7+ years. Children responded on a 3-point scale from *Never* to *Always*, describing events from the preceding month. The scale was adapted from an adult version (Opakunle et al., 2017) and shows robust test-retest reliability, concurrent validity with clinician-rated OCD symptom, as well as discriminant validity with anxiety symptoms (Foa et al., 2010). Foa et al. (2010) found total OCI-CV had an internal consistency of α = 0.85, and in our own sample we found an internal consistency of α = 0.93.

#### Creative Self-Concept

This measure was designed for this study to elicit children’s evaluation of their own creative ability. Creative self-concept is a robust indicator of creativity and correlates with direct measurements of creative activities and achievements in adults (McKay et al., 2017; Snyder et al., 2020). Since there are no similar scales for children (though ample literature showing self-concept *itself* is a reliable construct for children; e.g., in academic areas; Gao and Eccles, 2020) we created one for our purposes here. For this, we adapted an adult scale for creative self-concept (e.g., McKay et al., 2017) by shortening it to a two-item set for children, using language from child scales (of academic self-concept; e.g., Gao and Eccles, 2020). In the present study, children were therefore asked *How good are you at these subjects: Art/Music*? These items are key indicators of artistic creative concept (McKay et al., 2017; Snyder et al., 2020), and children were required to rate each one using a 7-point Likert scale, running from *1 = Not good at all* to *7 = Very good* (with the mid-point 4 marked as *Average*). We will average across items in our results, and note that they have an acceptable internal consistency (interitem correlation *r* = 0.25; *p* = 0.005).

## Results

### Identifying Children With Misophonia

In the adult questionnaire related to the scale used here (Rinaldi et al., 2021), scoring involves summing the 39 Likert-scale responses in Part 2^4^ (coded 0–4; giving a score out of 156), and comparing to the adult threshold for misophonia. The adult test has been used by several thousand misophonics to date, and Receiver Operator Characteristic show it to be an “excellent” measure for identifying misophonia in adults (Rinaldi et al., 2021). In children however, the threshold for misophonia is unknown. We therefore take a conservative approach by considering the prevalence of misophonia in adults (20%; Wu et al., 2014; see also Zhou et al., 2017) and conservatively applying half this prevalence to children, to set the child threshold at the 90th percentile of total SMS-Adolescent scores. This threshold captured all children with a test-score of 49 or higher, and we point out that this threshold is approximating the adult threshold on this scale (50.5; Rinaldi et al., 2021). Our conservative approach will allow us to be confident that we are identifying genuine child misophonics. (i.e., it aims to reduce false positives over false negatives).

Using this threshold score, we classified 15 children with misophonia. This group comprised nine girls (Mean age 11.67, SD 1.32) and six boys (Mean age 11.00, SD 0.89). The remaining 127 children were designated controls, and comprised 56 girls (Mean age 11.67, SD 1.22) and 71 boys (Mean age 11.83, SD 1.03). This relatively small sample has great value in being the first identified by screening of a population, rather than self-presenting at clinic. As such, they may represent an estimate of the children with misophonia in the population at large.

### Do Children With Misophonia Show Poorer Well-Being (in Anxiety, Obsessive Compulsive Disorder Traits, Health Related Quality of Life, and Satisfaction With Life)?

In our analyses, we first ran assumptions checks, which confirmed significant skews in our data across all measures. These skews are expected with well-being data, and reflect the fact that the majority of participants have no problems in their well-being, so their scores are at one end of the scale (e.g., within the Obsessive Compulsive Inventory, most participants will not have any obsessive compulsive symptoms and therefore score 0). To address this skew, we ran robust models where possible (following Field et al., 2012). As part of our assumptions checks we also screened for, and removed outliers by looking for *z*-scores above/below 3/−3. Instances where outliers were found are indicated below. We next ensured no violation of homogeneity of variance using Levene’s test, and we also include a variance ratio (where scores below 1.5 indicate no issues with homogeneity of variance; see Blanca et al., 2018). These tests are included below. We ran our group-wise analyses in *R* using “WRS2” for robust *t*-tests, and robust effect sizes using trimmed means. Given unequal sample sizes, a Hedges *g* correction may be applied. However our need for robust models combined with the fact that the robust effect sizes reported throughout are more conservative across the board, we report instead an explanatory measure of effect size ξ which holds the same interpretation as Cohen’s *d* (e.g., Values of ξ = 0.10, 0.30, and 0.50 correspond to small, medium, and large effect sizes respectively; Mair and Wilcox, 2020). We additionally used the R packages “afex” for ANOVA, “emmeans” for *post-hoc* estimated means tests, and “tidyverse” for general data wrangling.

**FIGURE 1:**
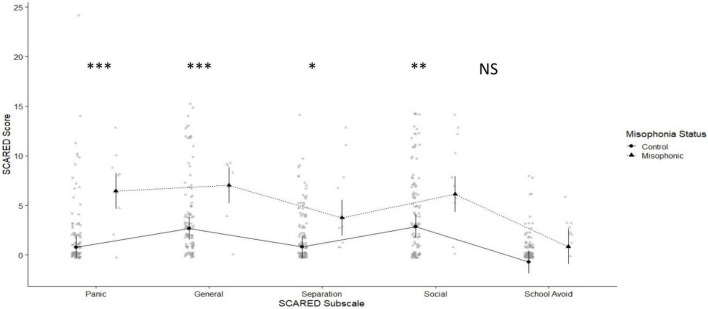
Means plot showing differences between misophonics (shown in triangle) and controls (shown in circles) in each of the SCARED subscales (from left to right: *Panic Disorder*, *General Anxiety*, *Separation Anxiety*, *Social Anxiety*, and *School Avoidance)*.

We first considered our parent-report questionnaire, for anxiety (the SCARED) where the maximum possible score is 82, and scores ≥ 25 may indicate the presence of an anxiety disorder (Birmaher et al., 1997, 1999). The overall mean for children with misophonia was 31.50 (SD 13.46) compared to 13.74 (SD 14.22) for controls. We found no problems with Levene’s test of homogeneity of variance [*F*_(1,113)_ = 0.003, *p* = 0.955; variance ratio 1.05]. We explored the SCARED in a 2×5 mixed ANOVA crossing group (misophonics vs. controls) with subscale (*Panic Disorder, General Anxiety, Separation Anxiety, Social Anxiety, and School Avoidance*; see Figure 1). We found a statistically significant main effect of group [*F*_(1,113)_ = 14.35, *p* < 0.001], and a significant but less interesting main effect of sub-scale [*F*_(3.32,374.74)_ = 20.59, *p* < 0.001, since scores are generally higher for some sub-scales over others]. We also found a significant interaction [*F*_(3.32,374.74)_ = 3.29, *p* = .020]. We ran *post-hoc* estimated marginal means tests to explore this interaction and found that misophonics were significantly higher across all SCARED subscales except for *School Avoidance* (where the numerical difference failed to reach significance; see Figure 1).

**FIGURE 2:**
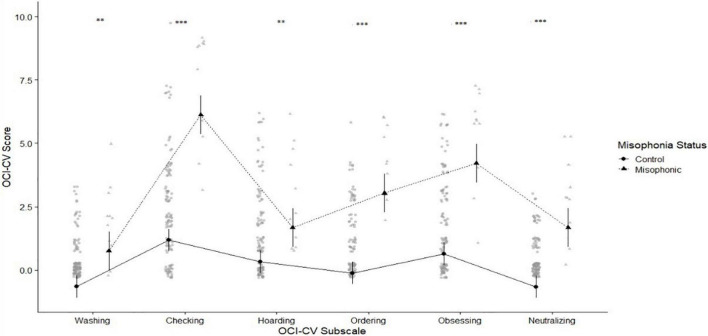
Means plot showing greater scores for misophonics (shown in triangle) and controls (shown in circles) in each of the OCI subscales (from left to right: *Washing*, *Checking and Doubting*, *Hoarding*, *Ordering*, *Obsessing*, and *Neutralizing*). Here and in all similar figures, means are shown with black circles/triangles, while grey points represent the raw data, with overlapping points appearing darker. Here and throughout, error bars show 95% confidence intervals, and the asterisks represent significant *p* values as follows: * < 0.05, ^**^ < 0.01, and ^***^ < 0.001.

We next considered our child-report measures, beginning with the OCI-CV for obsessive-compulsive traits (Foa et al., 2010). Mean scores for children with misophonia were 24.36 (SD 6.44) compared to controls who scored 7.63 (SD 6.59). We again found no problems with Levene’s [*F*_(1,122)_ = 0.64, *p* = 0.426; variance ratio 1.02] so we proceeded to explore the Obsessive Compulsive Inventory using a 2×6 mixed ANOVA crossing group (misophonics vs. controls) with subscale (*Washing*, C*hecking and Doubting, Hoarding, Ordering, Obsessing, and Neutralizing*; see Figure 2). We found a statistically significant main effect of group [*F*_(1,123)_ = 64.95, *p* < 0.001], a significant but less interesting main effect of sub-scale [*F*_(4.13,508.53)_ = 48.52, *p* < 0.001; since some sub-scales are higher than others], and a significant interaction [*F*_(4.13,508.53)_ = 13.19, *p* < 0.001]. We ran *post-hoc* estimated marginal means tests to explore this interaction and found misophonics had significantly higher obsessive compulsive traits across each subscale of the OCI (see Figure 2) but where differences are especially notable for *Neutralising* (ξ = 0.88, 95% CI 0.80–0.97), *Ordering* (ξ = 0.88, 95% CI 0.79–0.99), and *Obsessing* (ξ = 0.86, 95% CI 0.80–0.98).

We next considered health-related quality-of-life, and satisfaction with life, where scores are summed across items, and low scores correspond to poorer well-being. Within the *Very Short Well-being Questionnaire for Children* (VSWQ-C; Smees et al., 2020) we first ran our assumptions checks where we identified and removed three outliers, and confirmed that we had no problem with homogeneity of variance using Levene’s test [*F*_(1,137)_ = 0.06, *p* = 0.799; variance ratio 1.16]. We compared the mean score for children with misophonia 15.00 (SD 2.34) with controls 17.51 (SD 2.00). This difference was significant in a robust *t*-test (*t* (7.65) = 3.17, *p* = 0.001) with a large effect size (ξ = 0.69, 95% CI 0.55–0.83). We next looked at overall life satisfaction, (SWLS-C; Gadermann et al., 2010, 2011) where children with misophonia scored 13.77 (SD 4.28) compared to controls who scored 20.01 (SD 4.45). We again found no problems with Levene’s [*F*_(1,135)_ = 0.05, *p* = 0.821; variance ratio 1.03]. Again, the difference between misophonics and controls was significant (*t* (9.43) = 5.09, *p* < 0.001) with a large effect size (ξ = 0.78, CI 0.73–0.91).^5^

Table 2 shows that misophonia positively significantly correlated with obsessive-compulsive traits (OCI-CV; Foa et al., 2010) in Total and subscale scores, with all correlations surviving Bonferroni correction. Effects ranged from medium for the subscale *Hoarding* (*r* = 0.45, *p* < 0.001) to large for the Total score (*r* = 0.69, *p* < 0.001). We also found significant positive correlations between misophonia scores and anxiety (SCARED; Birmaher et al., 1999), in both total and subscale scores. These effects were moderate, ranging from *r* = 0.29 (*p* < 0.001) for *Social Anxiety*, to *r* = 0.43 (*p* < 0.001) for Total score. Finally, there was a significant and moderate negative correlation (*r* = −0.48, *p* < 0.001) between misophonia scores and health-related quality of life (VSWB-C; Smees et al., 2020). We also found a strong negative correlation between misophonia scores and satisfaction with life (SWLS-C; Gadermann et al., 2010, 2011; *r* = −0.56, *p* < 0.001). See Table 2 for a full list of these correlations, including with the subscales for anxiety and obsessive-compulsive traits.

**TABLE 2:** Spearman Correlations between misophonia scores and our remaining measures (*r* and *p* values) with 95% confidence intervals.

		Correlation with SMS-A (misophonia)		95% CI
	Subscale	*r* value	*p* value	
SCARED (Anxiety)	Total	0.43	<0.001	027–0.57
	General anxiety	0.38	<0.001	0.21–0.52
	Panic disorder	0.41	<0.001	0.24–0.55
	School avoidance	0.42	<0.001	0.26–0.55
	Separation anxiety	0.37	<0.001	0.20–0.51
	Social anxiety	0.29	<0.001	0.12–0.45

OCI-CV (Obsessive-compulsive)	Total	0.69	<0.001	0.47–0.69
	Washing	0.47	<0.001	0.30–0.58
	Checking/doubting	0.59	<0.001	0.51–0.72
	Hoarding	0.45	<0.001	0.50–0.72
	Neutralizing	0.62	<0.001	0.43–0.67
	Obsessing	0.62	<0.001	0.58–0.77
	Ordering	0.56	<0.001	0.33–0.60

VSWB	Total	−0.48	<0.001	−0.60 to −0.35

SWLS	Total	−0.56	<0.001	−0.67 to −0.44

Creative self-concept	Average creative self-concept	0.04	0.686	−0.16 – 0.21
	Art	0.07	0.406	−0.10 – 0.24
	Music	0.01	0.889	−0.16 – 0.19

### Do Children With Misophonia Show Differences in Creative Self-Concept?

Discriminant validity was assessed by considering scores in creative self-concept. As predicted, children with misophonia showed no differences in this area. Averaging across our two questions of creative self-construct (art, music), our assumptions checks showed non-normality. We therefore ran a robust *t*-test, however we found no problems with homogeneity of variance [Levene’s *F*_(1,127)_ = 0.04, *p* = 0.830; variance ratio 1.06]. As predicted, there were no significant differences between misophonics (Mean 4.29, SD 1.34) and controls (Mean 4.48, SD 1.26; *t* (8.41) = 0.17, *p* = 0.867) with a small effect size (ξ = 0.14, 95% CI 0.00–0.24). We explored our null result by producing a Bayes Factor to determine if there is enough evidence to accept the null hypothesis (Dienes, 2014). We found a JZS Bayes Factor of 0.329, where scores such as this (i.e., less than 1) provide evidence for the null hypothesis. Our Bayes passed the 0.33 threshold for moderate evidence. Similarly, a correlational approach shows an almost entirely non-existent relationship between misophonia scores and creative self-concept, with an *r* value of 0.01 (see Table 2).

## Discussion

In this study we examined – general population cohort of children with misophonia. These children showed significant differences compared to peers without misophonia. Primarily, they had higher traits associated with both anxiety disorder (SCARED; Birmaher et al., 1999) and obsessive-compulsive disorder (Foa et al., 2010). They also showed poorer health-related quality-of-life (in the VSWQ-C; Smees et al., 2020) and poorer satisfaction with life (Gadermann et al., 2010). Importantly, our screening for misophonia was child-completed, while at least one of our other measures was parent-completed (i.e., SCARED), meaning our results cannot be dismissed as a response bias (e.g., an acquiescence bias) since our data come from different individuals rating the same child.

Several previous studies have linked misophonia with anxiety/obsessive-compulsive traits (Schröder et al., 2013; Wu et al., 2014; Cusack et al., 2018; Naylor et al., 2020) and with poorer quality-of-life (Jager et al., 2020) – but importantly, only in adults. The current study extends this finding into children for the first time, and importantly, children in the population at large rather than those who have self-referred to treatment clinics (Our screening approach means we are almost certainly observing cases of misophonia that are likely to be as-yet unrecognised formally.). Prior to our study, there have been no validated measures to identify childhood misophonia. Here we have introduced our adolescent instrument the SMS-Adolescent, adapted from a related adults scale (Rinaldi et al., 2021). Our measure can be found in full in our appendix, as well as online at our website www.misophonia-hub.org/test where we provide an online interface and automated scoring. Our findings offer preliminary convergent validity for this scale, by showing it correlates with the related (but different) constructs of anxiety, obsessive-compulsive traits, life-satisfaction and health related well-being. This convergent validity has been particularly important in validating our measure given the lack of existing adolescent misophonia measures available for comparison (i.e., to offer concurrent validity; see Godwin et al., 2013 and Smees et al., 2020 for discussions on differences between convergent and concurrent validity). The strength of these convergent relationships ranged from moderate (for anxiety) to strong (for all the remainder), as we might expect from previous misophonia studies looking at similar characteristics in adults (e.g., Zhou et al., 2017). We also provided preliminary evidence of discriminant validation, by demonstrating that our measure of misophonia does not correlate with the unrelated construct of creative self-concept. We have necessarily applied our scale conservatively, identifying children in the 90th percentile and above. But future studies might validate our measure more widely on larger samples of adolescent misophonics to refine its threshold. A related goal is to also explore whether our measure has a factor structure, as it does in adults (see footnote 3).

The pattern of poor well-being we have identified in children with misophonia requires close attention. Adults studies (e.g., Wu et al., 2014) have suggested that misophonia is self-evidently related to anxiety and obsessive-compulsive disorders simply given its symptomatology (e.g., negative reactions triggered by sounds, associated anxiety and distress, and corresponding avoidance/compulsion). Here we tentatively suggest that obsessive-compulsive traits and misophonia may also be mediated by the factor of disgust. Disgust is a key emotional outcome of misophonia, but also shows important differences in OCD. Stein, Shapira, and colleagues have linked OCD to a disruption in disgust processing, with more inappropriate disgust compared to controls, and with disruptions mediated by the insula in both functional magnetic resonance imaging (fMRI; Shapira et al., 2003) and positron emission tomography (PET; Stein et al., 2006). This overlap between misophonia and OCD in both phenomenology and neural features may implicate disgust in their shared aetiology. We therefore suggest that future studies of misophonia may explore further the finding of elevated OCD-traits, shown both here in children, and elsewhere in adults.

These findings of poorer well-being in children with misophonia (e.g., heightened anxiety) raise the question of causality. We have assumed that misophonia may be responsible for our target children’s poorer well-being scores, although it is equally possible that children with poorer well-being (e.g., higher anxiety) may be more pre-disposed to developing misophonia. Of course these ideas are not mutually exclusive – and development will also be mediated by environment and genetics. One genetic marker for misophonia has been identified in a report by the organisation 23andMe (Fayzullina et al., 2015). They examined 80,607 participants who were asked “Does the sound of other people chewing fill you with rage?” (Yes/No/Not Sure). After removing responses of “Not Sure” and applying their criteria for genome-wide association significance.^6^ Fayzullina et al. found a significant genetic locus associated with misophonia – at least as far as they had phenotyped it with their single question. This locus, rs2937573 (chromosomal region 5q34), resides near the TENM2 gene, which encodes for the teneurin-2 protein, implicated in regulating synaptic connections during brain development (Vysokov et al., 2016; Tews et al., 2017). This finding supports evidence elsewhere of enhanced functional connectivity in misophonia (Kumar et al., 2017; Schröder et al., 2019). However, the four teneurin proteins also contain peptide sequences (teneurin C-terminal associated peptides; TCAP-1–4) which have been associated with anxiety behaviours in rats (Tan et al., 2009), and linked to structures implicated in other mood disorders (Woelfle et al., 2016). Future genetic studies may therefore hold the key for greater insight into the co-morbid relationship between misophonia and broader anxiety disorders.

We recognise that one limitation of our study is our small sample size, where our screening of 142 children identified 15 with misophonia. Hence, although our study presents promising data in support of the validity of our measure, this validation remains preliminary until future studies can replicate, and extend our findings on larger samples. We also point out that our cohort of 142 children were a sub-set from a much larger randomly-sampled cohort (MULTISENSE) but were not strictly randomly-sampled themselves (They were children whose parents had signed on for further study, comprising around 5% of the initial wave.). However, there were no well-being differences between our subset and the larger wave (using seven different well-being indicators, see footnote 1). This suggests our sample were indeed a meaningful reflection of the well-being of the entire random sample at large, and – furthermore – our misophonics and non-misophonics for the current study were recruited in exactly the same way (i.e., we look *within* this subset, based on a screening for misophonia). Nonetheless, future studies may wish to use our scales on larger random samples. Finally, our preliminary findings regarding divergent validity would benefit from replication using validated measures of creativity, and/or additional traits (so long as these traits are such that we would expect no convergence).

Our results begin to address a vacuum of knowledge concerning childhood misophonia, and highlight a need for further attention. We suggest that current and future research should promote actions to widen the public’s understanding of misophonia. Our data on well-being also suggest that professionals might engage in an active screening for anxiety disorder and obsessive-compulsions in any child where misophonia is suspected. At the same time, researchers and clinicians might push for a wider understanding of the condition in schools. One way to achieve this is to open dialogs between parents and teachers, where information about misophonia can be shared. To achieve this, we have created an online information hub^2^ as a one-stop resource containing advice and support for adults, children, parents, researchers, clinicians, and educators. The site also contains information factsheets about misophonia in both children and adults (e.g., our child factsheet is designed for parents to print, individualise, and share with their teacher). Feedback suggests our factsheets often provide well-needed validation for the “genuineness” of the child’s reports, because children with misophonia are often dismissed or disbelieved. Thus, impact has been at the heart of our research, and we propose a similar approach for future researchers. In summary, our study shows that misophonia can be identified in children aged 10–14 years, with negative implications for elevated anxiety and obsessive-traits, as well as poorer life satisfaction, and health-related quality of life.

## Data Availability Statement

The raw data supporting the conclusions of this article will be made available by the authors, without undue reservation.

## Ethics Statement

The studies involving human participants were reviewed and approved by Sussex University Science and Technology Ethics Committee (reference number ER/LR290/3). Written informed consent to participate in this study was provided by the participants’ legal guardian/next of kin.

## Author Contributions

LR: conceptualization, methodology, investigation, data curation, formal analysis, writing – original draft, visualization, and project administration. RS: writing – review and editing. JW: conceptualization, methodology, and funding acquisition. JS: conceptualization, methodology, resources, project administration, writing – original draft, review, and editing, and funding acquisition. All authors contributed to the article and approved the submitted version.

## Conflict of Interest

The authors declare that the research was conducted in the absence of any commercial or financial relationships that could be construed as a potential conflict of interest.

## Publisher’s Note

All claims expressed in this article are solely those of the authors and do not necessarily represent those of their affiliated organizations, or those of the publisher, the editors and the reviewers. Any product that may be evaluated in this article, or claim that may be made by its manufacturer, is not guaranteed or endorsed by the publisher.

## Footnotes

1 Our sample of 142 children was no different to the remainder in terms of a range of well-being metrics taken at earlier recruitment; i.e., not significantly different in positive affect [*t*(141.95) = 0.70, *p* = 0.49], negative affect [*t*(141.86) = 1.70, *p* = 0.09; for measure see Laurent et al., 1999], pro-social behaviour, emotional symptoms, conduct, hyperactivity, or peer problems (i.e., no effect of group [*F*_(1,534)_ = 0.79, *p* = 0.374], nor interaction [*F*_(3.21,1712.16)_ = 0.53, *p* = 0.673; for measure see Goodman, 2001]). Our sample were significantly higher than the remainder in spelling [*t*(149.21) = −6.04, *p* < 0.001] and math [*t*(113.88) = −7.16, *p* < 0.001] and this is perhaps to be expected from the children of parents who sign up for continued research (given the heritability of contentiousness and intelligence; Devlin et al., 1997; Luciano et al., 2006). The important point, however, is that our entire sample for the current study were recruited in exactly the same way, and we now look *within* this subset, based on a screening for misophonia. In other words, our sampling is likely to be unrelated to our findings on misophonia.

2
www.misophonia-hub.org

3 In the adult version of this questionnaire, a factor analyses by Rinaldi et al. (2021) revealed five factors, seen respectively in the five examples shown here, and these factors were: Feelings and Isolation (Items 2, 3, 4, 5, 10, 11, 16, 17, 18, 23, 24, 26, 27, 30, 32, and 38); Life Consequences (i.e., impact on work and friendships; Items 12, 13, 14, 21, 22, and 31); Intersocial Reactivity (Items 8, 15, 35, 36, and 37); Avoidance and Repulsion (Items 1, 7, 20, 28, 29, 33, 34, and 39); and Pain (suggestive of hyperacusis; Items 6, 9, 19, and 25). This factor analysis had reduced an original set of 53 items down to the 39 used here in the final version. Internal reliability of all factors was very high with Cronbach’s alpha estimates of 0.98, 0.94, 0.91, 0.92, and 0.95 for factors 1–5, respectively. However, we did not explore factor structure in adolescents because our sample size does not support this approach.

4 The comparable adult measure does not provide a score for Part 1 (triggers). Instead, it allows users to compare their own triggers against an ordered ranking. This ranking shows triggers listed from most to least common, according to a norming sample of ≈150 misophonic adults (Rinaldi et al., 2021). However, since this ordered ranking is not known for children, we omit this here. We therefore look to future studies where the nature of triggers for childhood misophonia can be better understood, and present our questionnaire in full here, for such purposes.

5 We considered a possible objection to our placing the threshold for misophonia at the 90th percentile (49 or higher; see above). We suggest this is an appropriate threshold because it is not only conservative (i.e., under-estimating prevalence) but closely equivalent to the adult threshold [50.5; shown statistically to be an “excellent” threshold; see Rinaldi et al. (2021)]. However, we also took a precautionary secondary approach, to consider the misophonia scale as a continuum and thereby re-analyse our measures using a correlational approach. To anticipate our results, we again found significance in all measures administered, mirroring our group-wise results above.

6
https://permalinks.23andme.com/pdf/23-08_genetic_associations_with_traits.pdf

## References

[B1] American Psychiatric Association (2013). *Diagnostic and Statistical Manual of Mental Disorders*, 5th Edn. Arlington, VA: American Psychiatric Association, 10.1176/appi.books.9780890425596

[B2] Ben-Arieh A. Frønes I. (2007). Indicators of Children’s well being: what should be measured and why? *Soc. Indic. Res.* 84 249–250. 10.1007/s11205-007-9183-6

[B3] Bird H. Franklin S. Howard D. (2001). Age of acquisition and imageability ratings for a large set of words, including verbs and function words. *Behav. Res. Methods Instrum. Comput.* 33 73–79. 10.3758/BF03195349 11296722

[B4] Birmaher B. Brent D. Chiappetta L. Bridge J. Monga S. Baugher M. (1999). Psychometric properties of the Screen for Child Anxiety Related Emotional Disorders (SCARED): a replication study. *J. Am. Acad. Child Adolesc. Psychiatry* 38 1230–1236. 10.1097/00004583-199910000-00011 10517055

[B5] Birmaher B. Khetarpal S. Brent D. Cully M. Balach L. Kaufman J. (1997). The Screen for Child Anxiety Related Emotional Disorders (SCARED): scale construction and psychometric characteristics. *Am. Acad. Child Adolesc. Psychiatry* 36 545–553. 10.1097/00004583-199704000-00018 9100430

[B6] Blanca M. J. Alarcón R. Arnau J. Bono R. Bendayan R. (2018). Effect of variance ratio on ANOVA robustness: might 1.5 be the limit? *Behav. Res. Methods* 50 937–962. 10.3758/S13428-017-0918-2/TABLES/17 28643157

[B7] Casas F. (2019). Introduction to the special section on Children’s subjective well-being. *Child Dev.* 90 333–343. 10.1111/cdev.13129 30102421

[B8] Chang L. R. Lin Y. H. Kuo T. B. J. Chang H. C. W. Liu C. M. Liu C. C. (2011). Autonomic modulation and health-related quality of life among schizophrenic patients treated with non-intensive case management. *PLoS One* 6:e26378. 10.1371/JOURNAL.PONE.0026378 22073161 PMC3208549

[B9] Cornaglia F. Crivellaro E. McNally S. (2015). Mental health and education decisions. *Labour Econ.* 33 1–12. 10.1016/J.LABECO.2015.01.005

[B10] Cusack S. E. Cash T. V. Vrana S. R. (2018). An examination of the relationship between misophonia, anxiety sensitivity, and obsessive-compulsive symptoms. *J. Obsessive Compuls. Relat. Disord.* 18 67–72. 10.1016/j.jocrd.2018.06.004

[B11] Davis C. J. (2005). N-Watch: a program for deriving neighborhood size and other psycholinguistic statistics. *Behav. Res. Methods* 37 65–70. 10.3758/BF03206399 16097345

[B12] Devlin B. Fienberg S. E. Resnick D. P. Roeder K. (eds) (1997). *Intelligence, Genes, and Success: Scientists Respond to the Bell Curve.* New York, NY: Springer Science & Business Media.

[B13] Diener E. (2000). Subjective well-being. The science of happiness and a proposal for a national index. *Am. Psychol.* 55 34–43. 10.1037/0003-066X.55.1.34 11392863

[B14] Diener E. Emmons R. A. Larsem R. J. Griffin S. (1985). The satisfaction with life scale. *J. Pers. Assess.* 49 71–75. 10.1207/s15327752jpa4901_13 16367493

[B15] Dienes Z. (2014). Using Bayes to get the most out of non-significant results. *Front. Psychol.* 5:781. 10.3389/FPSYG.2014.00781 25120503 PMC4114196

[B16] Dover N. McGuire J. F. (2021). Family-based cognitive behavioral therapy for youth with Misophonia: a case report. *Cogn. Behav. Pract.* (in press). 10.1016/J.CBPRA.2021.05.005

[B17] Eijsker N. Schröder A. Smit D. J. A. van Wingen G. Denys D. (2019). Neural basis of response bias on the stop signal task in misophonia. *Front. Psychiatry* 10:765. 10.3389/fpsyt.2019.00765 31708818 PMC6819955

[B18] Erhart M. Ottova V. Gaspar T. Jericek H. Schnohr C. Alikasifoglu M. (2009). Measuring mental health and well-being of school-children in 15 European countries using the KIDSCREEN-10 Index. *Int. J. Public Health* 54 160–166. 10.1007/s00038-009-5407-7 19652910

[B19] Fayzullina S. Smith R. P. Furlotte N. Hu Y. Hinds D. Tung J. Y. (2015). *White Paper 23-08 Genetic Associations with Traits in 23 and Me Customers*.

[B20] Fervaha G. Agid O. Takeuchi H. Foussias G. Remington G. (2016). Life satisfaction and happiness among young adults with schizophrenia. *Psychiatry Res.* 242 174–179. 10.1016/J.PSYCHRES.2016.05.046 27288735

[B21] Field A. Miles J. Field Z. (2012). *Discovering Statistics Using R.* Thousand Oaks, CA: Sage Publications.

[B22] Foa E. B. Coles M. Huppert J. D. Pasupuleti R. V. Franklin M. E. March J. (2010). Development and validation of a child version of the obsessive compulsive inventory. *Behav. Ther.* 41 121–132. 10.1016/j.beth.2009.02.001 20171333

[B23] Gadermann A. M. Guhn M. Zumbo B. D. (2011). Investigating the substantive aspect of construct validity for the satisfaction with life scale adapted for children: a focus on cognitive processes. *Soc. Indic. Res.* 100 37–60. 10.1007/s11205-010-9603-x

[B24] Gadermann A. M. Schonert-Reichl K. A. Zumbo B. D. (2010). Investigating validity evidence of the satisfaction with life scale adapted for children. *Soc. Indic. Res.* 96 229–247. 10.1007/s11205-009-9474-1

[B25] Gao Y. Eccles J. (2020). Who lower their aspirations? The development and protective factors of college-associated career aspirations in adolescence. *J. Vocat. Behav.* 116(Part A):103367. 10.1016/j.jvb.2019.103367

[B26] Gilhooly K. J. Logie R. H. (1980). Age-of-acquisition, imagery, concreteness, familiarity, and ambiguity measures for 1,944 words. *Behav. Res. Methods Instrum.* 12 395–427. 10.3758/BF03201693

[B27] Godwin M. Pike A. Bethune C. Kirby A. Pike A. Brunner E. (2013). Concurrent and convergent validity of the simple lifestyle indicator questionnaire. *ISRN Fam. Med.* 2013:529645. 10.5402/2013/529645 24967324 PMC4041224

[B28] Goodman R. (2001). Psychometric properties of the strengths and difficulties questionnaire. *Compr. Psychiatry* 40 1337–1345. 10.1097/00004583-200111000-00015 11699809

[B29] Jager I. de Koning P. Bost T. Denys D. Vulink N. (2020). Misophonia: phenomenology, comorbidity and demographics in a large sample. *PLoS One* 15:e0231390. 10.1371/journal.pone.0231390 32294104 PMC7159231

[B30] Jastreboff M. M. Jastreboff P. J. (2001). Components of decreased sound tolerance: hyperacusis, misophonia, phonophobia. *ITHS Newslett.* 1 1–5. 10.1055/s-0034-1372527

[B31] Jastreboff P. J. Jastreboff M. M. (2014). Treatments for decreased sound tolerance (Hyperacusis and Misophonia). *Semin. Hear.* 35 105–120. 10.1055/s-0034-1372527

[B32] Johnson P. L. Webber T. A. Wu M. S. Lewin A. B. Murphy T. K. Storch E. A. (2013). When selective audiovisual stimuli become unbearable: a case series on pediatric misophonia. *Neuropsychiatry* 3 569–575. 10.2217/NPY.13.70

[B33] Kamody R. C. del Conte G. S. (2017). Using dialectical behavior therapy to treat misophonia in adolescence. *Prim. Care Companion CNS Disord.* 19:17l02105. 10.4088/PCC.17L02105 28922587

[B34] Kılıç C. Öz G. Burcu K. Lu A. Aksoy Background S. (2021). The prevalence and characteristics of misophonia in Ankara, Turkey: population-based study. *BJPsych Open* 7 1–6. 10.1192/BJO.2021.978 34353403 PMC8358974

[B35] Kumar S. Tansley-Hancock O. Sedley W. Winston J. S. Callaghan M. F. Allen M. (2017). The brain basis for Misophonia. *Curr. Biol.* 27 527–533. 10.1016/j.cub.2016.12.048 28162895 PMC5321671

[B36] Laurent J. Catanzaro S. J. Joiner T. E. Rudolph K. D. Potter K. I. Lambert S. (1999). A measure of positive and negative affect for children: scale development and preliminary validation. *Psychol. Assess.* 11 326–338. 10.1037/1040-3590.11.3.326

[B37] Lewin A. B. Dickinson S. Kudryk K. Karlovich A. R. Harmon S. L. Phillips D. A. (2021). Transdiagnostic cognitive behavioral therapy for misophonia in youth: methods for a clinical trial and four pilot cases. *J. Affect. Disord.* 291 400–408. 10.1016/J.JAD.2021.04.027 34001373

[B38] Lindeboom M. van den Berg G. von Hinke Kessler Scholder S. Washbrook E. (2010). “Child mental health problems and youth educational attainment in the UK: evidence from the Avon Longitudinal Study of Parents and Children,” in *Proceedings of the Conference of Epidemiological Longitudinal Studies in Europe (CELSE)*, Pathos: CELSE.

[B39] Luciano M. Wainwright M. A. Wright M. J. Martin N. G. (2006). The heritability of conscientiousness facets and their relationship to IQ and academic achievement. *Pers. Individ. Differ.* 40 1189–1199. 10.1016/j.paid.2005.10.013

[B40] Mair P. Wilcox R. (2020). Robust statistical methods in R using the WRS2 package. *Behav. Res. Methods* 52 464–488. 10.3758/s13428-019-01246-w 31152384

[B41] McKay A. S. Karwowski M. Kaufman J. C. (2017). Measuring the muses: validating the Kaufman Domains of Creativity Scale (K-DOCS). *Psychol. Aesthet. Creat. Arts* 11 216–230. 10.1037/ACA0000074

[B42] McLellan R. Steward S. (2015). Measuring children and young people’s wellbeing in the school context. *Camb. J. Educ.* 45 307–332. 10.1080/0305764X.2014.889659

[B43] Mehdi T. Ahmadi N. (2011). Kernel smoothing For ROC curve and estimation for thyroid stimulating hormone. *Int. J. Public Health Res. Special Issue* 2011 239–242.

[B44] Morrison Gutman L. Vorhaus J. (2012). *The Impact of Pupil Behaviour and Wellbeing on Educational Outcomes Childhood Wellbeing Research Centre.* London: Department for Education.

[B45] Naylor J. Caimino C. Scutt P. Hoare D. J. Baguley D. M. (2020). The prevalence and severity of Misophonia in a UK undergraduate medical student population and validation of the Amsterdam Misophonia Scale. *Psychiatr. Q.* 90 609–619. 10.1007/s11126-020-09825-3 32829440 PMC8110492

[B46] Newland L. A. Giger J. T. Lawler M. J. Roh S. Brockevelt B. L. Schweinle A. (2019). Multilevel analysis of child and adolescent subjective well-being across 14 countries: child- and country-level predictors. *Child Dev.* 90 395–413. 10.1111/cdev.13134 30171770

[B47] Opakunle T. Akinsulore A. Aloba O. O. Fatoye F. O. (2017). Obsessive–compulsive symptoms in schizophrenia: prevalence and associated factors in a Nigerian population. *Int. J. Psychiatry Clin. Pract.* 21 195–200. 10.1080/13651501.2017.1330417 28554299

[B48] Palumbo D. B. Alsalman O. de Ridder D. Song J. J. Vanneste S. (2018). Misophonia and potential underlying mechanisms: a perspective. *Front. Psychol.* 9:953. 10.3389/fpsyg.2018.00953 30008683 PMC6034066

[B49] Parry-Langdon N. Clements A. Fletcher D. Goodman R. (2008). *Three Years on: Survey of the Development and Emotional Well-Being of Children and Young People.* Newport: Office for National Statistics.

[B50] Pollard E. L. Lee P. D. (2003). Child well-being: a systematic review of the literature. *Soc. Indic. Res.* 61 59–78. 10.1023/A:1021284215801

[B51] Potgieter I. MacDonald C. Partridge L. Cima R. Sheldrake J. Hoare D. J. (2019). Misophonia: a scoping review of research. *J. Clin. Psychol.* 75 1203–1218. 10.1002/JCLP.22771 30859581

[B52] Ravens-Sieberer U. KIDSCREEN Group Europe (2006). *The Kidscreen Questionnaires: Quality of Life Questionnaires for Children and Adolescents; Handbook.* Lengerich: Pabst Science Publishers.

[B53] Rinaldi L. J. Ward J. Simner J. (2021). An automated online assessment for Misophonia: the Sussex Misophonia scale for adults. *PsyArXiv* [Preprint] 10.31234/osf.io/5eb39 PMC11528938 38414185

[B54] Rosenthal M. Z. Anand D. Cassiello-Robbins C. Williams Z. J. Guetta R. E. Trumbull J. (2021). Development and initial validation of the duke Misophonia questionnaire. *Front. Psychol.* 12:4197. 10.3389/FPSYG.2021.709928/BIBTEX PMC8511674 34659024

[B55] Rouw R. Erfanian M. (2018). A large-scale study of Misophonia. *J. Clin. Psychol.* 74 453–479. 10.1002/jclp.22500 28561277

[B56] Ryff C. D. Lee C. Keyes M. (1995). The structure of psychological well-being revisited. *J. Pers. Soc. Psychol.* 69 719–727. 10.1037/0022-3514.69.4.719 7473027

[B57] Sammons P. Sylva K. Melhuish E. C. Siraj-Blatchford I. Taggart B. Jelicic H. (2008). *Relationships Between Pupils’ Self-Perceptions, Views of Primary School and Their Development in Year 5.* London: Institute of Education.

[B58] Schröder A. Vulink N. Denys D. (2013). Misophonia: diagnostic criteria for a new psychiatric disorder. *PLoS One* 8:e54706. 10.1371/journal.pone.0054706 23372758 PMC3553052

[B59] Schröder A. Wingen G. van Eijsker N. San Giorgi R. Vulink N. C. Turbyne C. (2019). Misophonia is associated with altered brain activity in the auditory cortex and salience network. *Sci. Rep.* 9:7542. 10.1038/s41598-019-44084-8 31101901 PMC6525165

[B60] Shapira N. Liu Y. He A. Bradley M. Lessig M. James G. (2003). Brain activation by disgust-inducing pictures in obsessive-compulsive disorder. *Biol. Psychiatry* 54 751–756. 10.1016/S0006-3223(03)00003-9 14512216

[B61] Simner J. Smees R. Rinaldi L. J. Carmichael D. A. (2021). Wellbeing differences in children with synaesthesia: anxiety and mood regulation. *Front. Biosci. (Elite Ed.)* 13:195–215. 10.2741/878 33048782

[B62] Smees R. Rinaldi L. J. Simner J. (2020). Well-being measures for younger children. *Psychol. Assess.* 32 154–169. 10.1037/pas0000768 31599610

[B63] Snyder H. T. Sowden P. T. Silvia P. J. Kaufman J. C. (2020). The creative self: do people distinguish creative self- perceptions, efficacy, and personal identity? *Psychol. Aesthet. Creat. Arts* 15 627–636. 10.1037/ACA0000317

[B64] Solans M. Pane S. Estrada M.-D. Serra-Sutton V. Berra S. Herdman M. (2008). Health-related quality of life measurement in children and adolescents: a systematic review of generic and disease-specific instruments. *Value Health* 11 742–764. 10.1111/j.1524-4733.2007.00293.x 18179668

[B65] Stein D. J. Arya M. Pietrini P. Rapoport J. L. Swedo S. E. (2006). Neurocircuitry of disgust and anxiety in obsessive-compulsive disorder: a positron emission tomography study. *Metab. Brain Disease* 21 267–277. 10.1007/S11011-006-9021-6 16850255

[B66] Swedo S. Baguley D. M. Denys D. Dixon L. J. Erfanian M. Fioretti A. (2022). A consensus definition of misophonia: using a delphi process to reach expert agreement. *Front. Neurosci.* (in press). 10.3389/fnins.2022.841816 PMC8969743 35368272

[B67] Sylva K. Melhuish E. Sammons P. Siraj-Blatchford I. Taggart B. Hunt S. (2008). *Effective Pre-School and Primary Education 3–11 Project (EPPE 3–11): Final Report From the Primary Phase: Pre-School, School and Family Influences on Children’s Development During Key Stage 2 (7–11).*

[B68] Tan L. A. Xu K. Vaccarino F. J. Lovejoy D. A. Rotzinger S. (2009). Teneurin C-terminal associated peptide (TCAP)-1 attenuates corticotropin-releasing factor (CRF)-induced c-Fos expression in the limbic system and modulates anxiety behavior in male Wistar rats. *Behav. Brain Res.* 201 198–206. 10.1016/j.bbr.2009.02.013 19428634

[B69] Taylor C. (2018). The reliability of free school meal eligibility as a measure of socio-economic disadvantage: evidence from the Millennium Cohort study in Wales. *Br. J. Educ. Stud.* 66 29–51. 10.1080/00071005.2017.1330464

[B70] Tews D. Fromme T. Keuper M. Hofmann S. M. Debatin K. M. Klingenspor M. (2017). Teneurin-2 (TENM2) deficiency induces UCP1 expression in differentiating human fat cells. *Mol. Cell. Endocrinol.* 443 106–113. 10.1016/j.mce.2017.01.015 28088466

[B71] The Whoqol Group (1998). The World Health Organization quality of life assessment (WHOQOL): development and general psychometric properties. *Soc. Sci. Med.* 46 1569–1585. 10.1016/S0277-9536(98)00009-4 9672396

[B72] Vitoratou S. Uglik-Marucha N. Hayes C. Gregory J. (2021). Listening to people with Misophonia: exploring the multiple dimensions of sound intolerance using a new psychometric tool, the S-Five, in a large sample of individuals identifying with the condition. *Psych* 3 639–662. 10.3390/psych3040041

[B73] Vysokov N. V. Silva J. P. Lelianova V. G. Ho C. Djamgoz M. B. Tonevitsky A. G. (2016). The mechanism of regulated release of lasso/teneurin-2. *Front. Mol. Neurosci.* 9:59. 10.3389/fnmol.2016.00059 27499734 PMC4956664

[B74] Webber T. A. Johnson P. L. Storch E. A. (2014). Pediatric misophonia with comorbid obsessive-compulsive spectrum disorders. *Gen. Hosp. Psychiatry* 36 231.e1–231.e2. 10.1016/j.genhosppsych.2013.10.018 24333158

[B75] Williams Z. J. He J. L. Cascio C. J. Woynaroski T. G. (2021). A review of decreased sound tolerance in autism: definitions, phenomenology, and potential mechanisms. *Neurosci. Biobehav. Rev.* 121 1–17. 10.1016/J.NEUBIOREV.2020.11.030 33285160 PMC7855558

[B76] Woelfle R. D’Aquila A. L. Lovejoy D. A. (2016). Teneurins, TCAP, and Latrophilins: roles in the etiology of mood disorders. *Transl. Neurosci.* 7 17–23. 10.1515/tnsci-2016-0004 28123817 PMC5017594

[B77] World Health Organization (2020). *International Statistical Classification of Diseases and Related Health Problems*, 11th Edn. Geneva: World Health Organization.

[B78] Wu M. S. Lewin A. B. Murphy T. K. Storch E. A. (2014). Misophonia: incidence, phenomenology, and clinical correlates in an undergraduate student sample. *J. Clin. Psychol.* 70 994–1007. 10.1002/jclp.22098 24752915

[B79] Zhou X. Wu M. S. Storch E. A. (2017). Misophonia symptoms among Chinese university students: incidence, associated impairment, and clinical correlates. *J. Obsessive Compuls. Relat. Disord.* 14 7–12. 10.1016/j.jocrd.2017.05.001

