# Supplementary material for: Poorer Well-Being in Children With Misophonia: Evidence From the Sussex Misophonia Scale for Adolescents
Source: Front Psychol. 2022 Apr 6;13:808379. doi: 10.3389/fpsyg.2022.808379 (PMC9019493; doi:10.3389/fpsyg.2022.808379)
Supplement: Supplementary file 1 [file Presentation_1.pdf]

Appendix: The Sussex Misophonia Scale for Adolescents (SMS-Adolescent)

This test is available online with automatic scoring at www.misophonia-hub.org/test

Part 1

We're going to ask you about things you see and hear every day. Have you always hated these things? Or don't you mind them? I hate… *(categories 1-8 are shown on screen; with sub-scale items revealed in the event of a positive response to the category)*

1 the sound of people eating yes/no
Which do you hate hearing? Tick all that apply.
crunchy foods (e.g. apples); crispy snacks; chewing; lip smacking; swallowing; slurping (a drink); wet mouth sounds (e.g., yoghurt); other

2 the sound of repetitive tapping yes/no
Which do you hate hearing? Tick all that apply.
pen clicking; foot tapping/ foot on floor; repetitive barking; tapping pen/ pencil; tapping finger; typing on a computer; other

3 the sound of rustling yes/no
Which do you hate hearing? Tick all that apply.
rustling paper; rustling plastic; other

4 throat sounds yes/no
Which do you hate hearing? Tick all that apply.
throat clearing; hiccups; humming; other

5 sounds people make through their mouth and nose yes/no
Which do you hate hearing? Tick all that apply.
breathing; snorting (e.g., when people laugh); nose sniffing; coughing; snoring; whistling; sneezing; burping; other

6 some voice sounds yes/no
Which do you hate hearing? Tick all that apply.
certain accents; some people's voices; certain letter sounds; certain vowels; certain consonants; other

7 repetitive visual movements yes/no
Which do you hate seeing? Tick all that apply.
repetitive leg rocking; foot shuffling; people rocking back and forth on their chair; other

8 some background sounds (e.g., fridge humming) yes/no
Which do you hate hearing? Tick all that apply.
clock ticking; car engines; refrigerator humming; dishwasher; washing machine/ dryer; fan; other

Part 2

How often do these things happen to you? (Likert responses: Never, Hardly ever, Sometimes, Often, Always).

1. Certain sounds make me feel disgusted, even if those sounds don't disgust other people.
2. I have a problem because hearing certain sounds makes me unhappy.
3. I feel no one really understands that I have a problem with sounds.
4. I feel scared hearing sounds I don’t like.
5. Other people make fun of me for hating sounds.
6. It hurts when I hear certain sounds, even if it doesn't hurt other people.
7. Sometimes I leave the room, to avoid telling people off for making bad sounds.
8. There are some sounds I hate so much, I shout at people.
9. Sounds often cause me physical pain.
10. I feel guilty because of my reaction to sounds.
11. I worry nobody can help with my sound problems.
12. I don't do well at school because of distractions from sounds.
13. I try to avoid going to people's houses if those people make sounds I hate.
14. I try to avoid going to school because of difficulties with sounds.
15. I hate people who make sounds I don't like.
16. I feel embarrassed about hating certain sounds.
17. Nobody believes my problem with sounds.
18. Hatred of some sounds make me feel lonely.
19. I feel physical pain if unable to avoid a sound.
20. The sound made by some people makes me feel the need to avoid them.
21. Problems with sounds has meant I don't have many friends.
22. My hatred of some sounds creates problems in school.
23. I'm worried about always having problems from hearing certain sounds.
24. I try not to let people know I hate certain sounds.
25. I feel pain on my body when I hear certain sounds.
26. My life is worse because of sound problems.
27. People think I'm crazy because of my reaction to sounds.
28. I cover my ears to block out certain sounds.
29. I've told some people they must not make certain noises around me.
30. Some sounds make me want to scream or cry.
31. I don't like school because there are lots of sounds I hate.
32. I suspect my friends think I'm weird, because of my reaction to sounds.
33. I react more strongly to some sounds if I'm having a bad day.
34. I say things aloud in order to avoid listening to bad sounds.
35. I want to hurt people who make sounds I hate.
36. I feel like people make sounds on purpose just to upset me.
37. I want to get pay-back on people who make certain sounds.
38. I think my problems with sounds are getting worse with age.
39. I put on headphones to block out certain sounds.

Scoring

Part 1. This section does not form part of the misophonia diagnosis, but researchers may wish to use this section for descriptive statistics (e.g., describing the number of triggers reported by their participant) or related analyses.

Part 2. Likert responses are scored 0-4 (0-never; 1-hardly ever; 2-sometimes; 3-often; 4-always) and are then summed to give scores running from 0-156, where the passing threshold to indicate misophonia from this study was 49 or higher.
